# Influence of Depth of Interaction upon the Performance of Scintillator Detectors

**DOI:** 10.1371/journal.pone.0098177

**Published:** 2014-05-29

**Authors:** Mark S. Brown, Stefan Gundacker, Alaric Taylor, Clemens Tummeltshammer, Etiennette Auffray, Paul Lecoq, Ioannis Papakonstantinou

**Affiliations:** 1 Electrical and Electronic Engineering, UCL, London, United Kingdom; 2 PH-CMX Group, CERN, Geneva, Switzerland; Helmholtz-Zentrum Dresden-Rossendorf, Germany

## Abstract

The uncertainty in time of particle detection within a scintillator detector, characterised by the coinci- dence time resolution (CTR), is explored with respect to the interaction position within the scintillator crystal itself. Electronic collimation between two scintillator detectors is utilised to determine the CTR with depth of interaction (DOI) for different materials, geometries and wrappings. Significantly, no rela- tionship between the CTR and DOI is observed within experimental error. Confinement of the interaction position is seen to degrade the CTR in long scintillator crystals by 10%.

## Introduction

**Figure 1:**
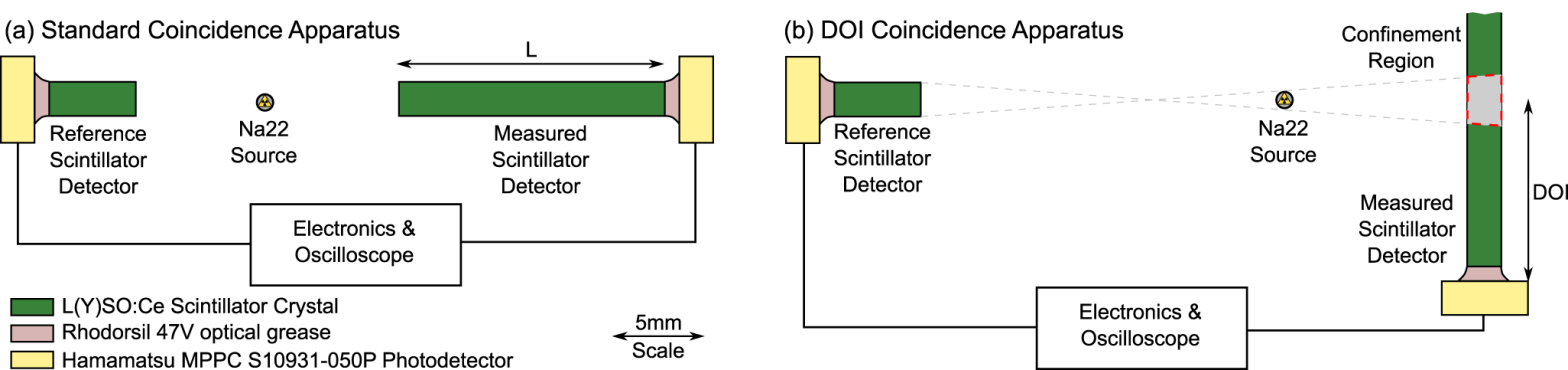
The two timing coincidence apparatuses. The two timing coincidence apparatuses. In (a) the standard coincidence apparatus, used in this paper to measure the timing performance with the scintillator crystal length, *L* is shown. In (b) the depth of interaction (DOI) coincidence apparatus is seen. In this, the measured scintillator detector is rotated 90 degrees, with respect to the reference scintillator detector. In the standard coincidence apparatus the Na22 source is placed equidistant between the two scintillator detectors, whereas in the DOI coincidence apparatus the source is placed much closer to the measured scintillator detector. This leads to electronic collimation forming a confinement region within the measured scintillator detector. The confinement region is shown in grey surrounded by a red dashed line [1].

Detection of ionising radiation is typically accomplished by transducing the incoming particle into light. This light can then be converted to an electrical signal and subsequently analysed. Scintillator detectors are comprised of three primary components, as shown in figure 1. Namely a scintillator crystal for creation of thousands of optical photons, a photodetector for conversion of the light to an electrical signal and a layer of optical grease between the two components to improve coupling. Reductions in the scintillator detector detection time uncertainty, known as the time resolution, are important for reducing statistical noise in positron emission tomography (PET) images [1].

In this work we investigate the relationship between the interaction position of 0.511 MeV gamma ray photons and the timing and energy performance of the scintillator detector. The depth of interaction (DOI), shown in figure 1, is the shortest distance to the photodetector from the gamma ray photon (*γ*) interaction position. The DOI is a potential source of degradation to the timing and energy performance of the scintillator detector due to photon time of flight and light loss from increased path lengths within the scintillator crystal. Furthermore determination of the DOI, of a given interaction, is of importance for PET to negate or reduce the contribution of parallax error upon the spatial resolution [2] [3]. If successful, longer scintillator crystals may be used leading to an improvement in the PET scanner's sensitivity and reduce overall scan times. Within monolithic scintillator detectors the same DOI information allows spatial confinement within the detector itself [4] [5], thus potentially allowing more novel [6] [7] layouts and geometries.

In this paper we begin by describing the standard and DOI coincidence apparatus, along with the method utilised in both for analysing the raw data in section. Using this method we characterise the 2×2×5 mm^3^ Agile Ca-co-doped LSO:Ce scintillator crystal used in the reference scintillator detector in section. Once this accomplished the time resolution with scintillator crystal length (*L*) is explored with the standard coincidence apparatus using two identical 2×2× *L* mm^3^ Proteus LYSO:Ce scintillator crystals in section. Measurements conducted using the DOI coincidence apparatus are split into two. Firstly for two identical 2×2×30 mm^3^ Proteus LYSO:Ce and secondly for a single 2×2×20 mm^3^ Agile Ca-co-doped LSO:Ce. These are covered in sections and respectively. In doing so we explore the contribution, if any, of scintillator crystal material, geometry and wrapping. All scintillator crystals are polished. PTFE (Teflon) tape is used as the wrapping material due its diffusive properties. Finally, we discuss the results in the discussion in section.

## Method

### Overview

The timing coincidence apparatus used in this paper is comprised of two Hamamatsu MPPC S10931-050P SiPMs connected to CERN-developed NINO leading-edge discriminators via analogue amplifiers. The energy and timing information of individual pulses are collected using a LeCroy DDA 735Zi high- bandwidth oscilloscope. Our coincidence apparatus is held within a temperature-controlled chamber to maintain stability of photodetector performance. The first 5 minutes of each measurement are discarded due to any potential contribution of temperature variation.

Scintillator crystals are coupled to the SiPM photodetectors using Rhodorsil 47 V optical grease to improve light output. The refractive indices of L(Y)SO:Ce and the optical grease are approximately 1.8 [8] and 1.4 [9] respectively. Wrapped scintillator crystals are tightly bound in many layers of PTFE tape to ensure good coupling between the scintillator crystal and wrap. Prior to wrapping and usage, all scintillator crystals are cleaned using isopropyl alcohol. All scintillator crystals are handled with carbon-tipped tweezers to prevent formation of surface defects which may degrade the scintillator crystal performance.

The optimal threshold and bias values of the SiPMs were determined by parameter sweep and are given in table 1. A thorough description of the experimental method can be found in [10].

**Table 1:** Main SiPM parameters used for standard and DOI timing coincidence measurements.

	Bias (V)	Overvoltage (V)	Threshold (V)
Left photodetector	72.6	2.2	1.64, 1.56
Right photodetector	72.7	2.2	1.64, 1.56

### Processing Data

The positron emission from the Na22 source will generate two 0.511 MeV gamma ray photons in opposition correlated in time. By selecting for events which interact solely by the photoelectric effect we ensure that the incident gamma ray photon has interacted with matter only once. Therefore if two gamma ray photons are detected in opposition within a small time window, it is highly likely they are from the same electron-positron annihilation. It is this ‘electronic collimation’ timing property which ensures we only record events from within the confinement region. These events are found by selecting the subset of interactions which fall within 2*σ* of the photopeak centroid of their respective energy spectra. This narrow range is chosen to drastically reduce the contribution of overlapping Compton interactions. When two gamma ray photons are detected within their respective photopeak energy ranges, within a nanosecond of each other, the relative time delay between the two is recorded. For many such true events the relative difference in arrival time is histogrammed to produce a Gaussian distribution. This will be referred to as the (relative) delay peak. For two identical photodetectors the FWHM of the delay peak is defined as the coincidence time resolution (CTR), such that
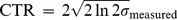
(1)




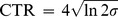
(2)where 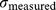 is the scale parameter measured from the delay peak and *σ* is the time resolution of the scintillator detector. This relationship holds because the delay peak is formed from the convolution of two Gaussian distributions, correponding to the delay peaks of the individual scintillator detectors. In cases where we use a reference scintillator detector with a known time resolution, the CTR of an unknown scintillator detector is determined by subtraction in quadrature and a subsequent scaling such that



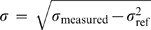
(3)

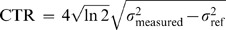
(4)where 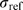 is the (known) reference time resolution. All CTR values in this paper are given in picoseconds.

### Analysis of Data

The parameters describing the location and scale of the Gaussian distributions (the photopeaks and delay peak per measurement) were found by weighted least-squared fit. The error per bin was assumed Poissonian and taken as the square root of the number of measurements per bin. The standard error in the fit parameters were determined by the bootstrap method [11]. The full code used to perform the peak detection, peak fitting, parameter error determination and image & table generation can be found at https://github.com/marksbrown/ProcessingCTRData. An online version of this paper can be found at [12].

## Results

### Reference Detector Measurements

**Figure 2:**
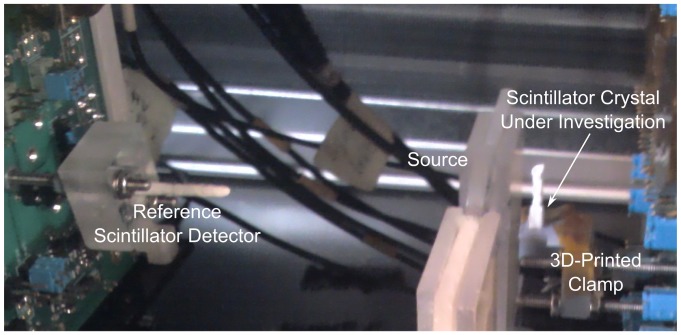
Photograph of the DOI timing coincidence apparatus. The timing coincidence apparatus set up for DOI measurements. The reference detector, shown on the left of the image, is a 2×2×5 mm^3^ Agile Ca-co-doped LSO:Ce wrapped in PTFE tape coupled to a Hamamatsu MPPC S10931-050P SiPM using Rhodorsil 47 V optical grease. On the right, the source is shown close to the 3D-printed clamp holding the same photodetector coupled to the scintillator crystal under investigation.

The reference scintillator crystal, shown on the left of figure 2, is a 2×2×5 mm^3^ Agile Ca-co-doped LSO:Ce wrapped in PTFE tape. Using two such identical crystals the CTR was determined using both the standard and DOI coincidence apparatuses. The key values from the measurements are shown in table 2 as time resolution and as CTR values. We note that the CTR from both measurements are in agreement. The CTR value from the standard coincidence measurement is in agreement with a prior measurement in [Table 2] [13] of 123±7 ps. In this paper the reference detector CTR value is taken as 131±4 ps unless otherwise stated.

**Table 2:** Coincidence time resolution values for two identical polished 2×2×5 mm^3^ Ca-co-doped LSO:Ce wrapping in PTFE tape for standard and DOI measurements.

Coincidence Apparatus	Left Energy Resolution (%)	Right Energy Resolution (%)	Detected *γγ* Events	Valid *γγ Events*	Delay Peak Centroid (ps)	σ_ref_ (ps)	CTR (ps)
Standard	7.8±0.1	9.8±0.1	8533±92	637±25	−77.5±2.3	39.3±1.2	131.0±3.9
DOI	10.5±0.1	12.7±0.1	18894±137	1498±39	−14.0±1.6	39.6±0.9	132.0±3.0

### Standard Coincidence

**Figure 3:**
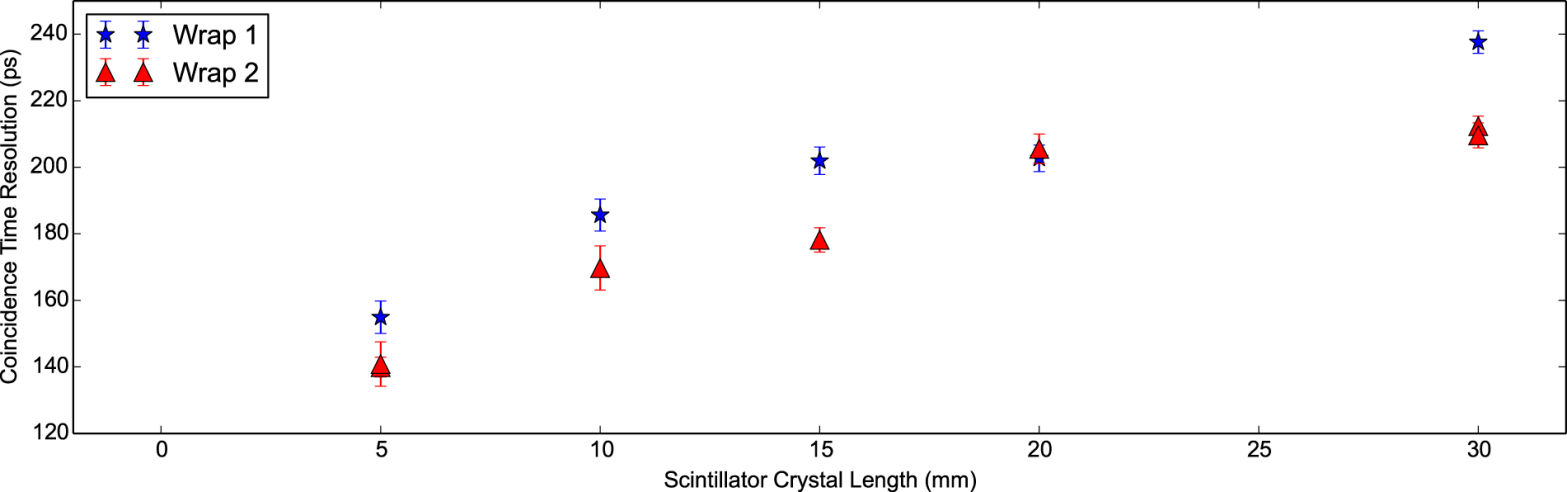
CTR against scintillator crystal length. The CTR with scintillator crystal length is plotted for Proteus LYSO:Ce scintillator crystals of lengths 5, 10, 15, 20 and 30 mm. All crystals possess a cross section of 2×2 mm^2^.

Prior to the DOI experiment, standard coincidence measurements are made of Proteus LYSO:Ce scin-tillator crystals with a cross section of 2×2 mm^2^ wrapped in PTFE tape for lengths, *L*, of 5, 10, 15, 20 and 30 mm. Two identical crystals, which are referred to as *L*A and *L*B, of each length are placed into opposing, identical, scintillator detectors. The CTR is then determined as the FWHM of the delay peak directly. Furthermore electronic collimation ensures the confinement region is the same in both scintillator crystals. In figure 3 we see the expected degradation of the CTR with increasing crystal length [14] [15] [16] [17] [18]. The full set of results are given in table 3. In these measurements the wrapping was reapplied by a second individual and the measurement retaken. These will referred to as ‘wrap 1’ and ‘wrap 2’. For 5 mm and 30 mm the measurement is repeated by the same person. We see that the ‘quality’ of the wrapping leads to a large systematic variation that must be carefully taken into account for measurements to be validly compared between scintillator crystals. Given that we also see a shift in the delay peak centroid between these measurements, we would conclude correct alignment upon the SiPM is also vital.

**Table 3:** Standard coincidence apparatus measurements for two identical polished Proteus LYSO:Ce scintillator crystals wrapped in PTFE tape.

Wrapped By	length (mm)	Left Energy Resolution (%)	Right Energy Resolution (%)	Detected *γγ* Events	Valid *γγ Events*	Delay Peak Centroid (ps)	CTR (ps)
Wrap 1	5	7.05±0.07	9.44±0.09	856±29	11177±106	−68.8±2.4	154.9±4.9
Wrap 2	5	6.33±0.08	7.36±0.09	361±19	6344±80	−68.8±3.4	140.8±6.7
Wrap 2	5	6.89±0.05	7.29±0.05	1526±39	19359±139	−64.8±1.6	139.9±3.0
Wrap 1	10	8.24±0.06	9.63±0.07	1032±32	18224±135	−124.4±2.5	185.6±4.8
Wrap 2	10	6.66±0.05	7.59±0.05	515±23	21032±145	−168.4±3.6	169.7±6.6
Wrap 1	15	9.05±0.05	9.07±0.05	1875±43	30728±175	−176.0±2.0	201.9±4.1
Wrap 2	15	6.82±0.04	7.67±0.04	1569±40	29562±172	−108.8±2.0	178.2±3.6
Wrap 1	20	10.97±0.05	9.85±0.06	1731±42	34350±185	−85.2±2.2	202.7±4.0
Wrap 2	20	6.99±0.03	9.27±0.03	2003±45	66796±258	−131.1±2.2	205.5±4.4
Wrap 1	30	10.94±0.05	13.07±0.06	3382±58	38913±197	−58.6±1.8	237.7±3.4
Wrap 2	30	9.73±0.04	10.79±0.05	3502±59	39872±200	−70.6±1.6	212.4±3.0
Wrap 2	30	9.52±0.05	10.72±0.06	2369±49	26741±164	−67.9±1.9	209.6±3.7

All crystals have a cross section of 2×2 mm^2^.

Also in table 3 we see that the energy resolution (%), for both left and right scintillator detectors, is poorer at higher scintillator crystal lengths. This is due to increased variance in the energy recorded and reduced light detected for a 0.511 MeV gamma ray photon; likely due to increased path length of photons through the scintillator crystal. Also note that each measurement was conducted for 15 minutes each. Thus the number of *γγ* events detected increases with the scintillator crystal lengths as expected due to greater volume within the confinement region, leading to increased sensitivity.

### Depth Of Interaction Coincidence

The standard coincidence apparatus, as shown in figure 1(a) is altered in two key respects. Firstly the right photodetector is placed within a 3D-printed clamp designed to hold the scintillator crystal which is held vertically with respect to the reference detector. Secondly the ^22^Na source is placed much closer to the vertically aligned scintillator detector than the reference. As in the standard apparatus both scintillator crystals are coupled to their respective photodetectors, Hamamatsu MPPC S10931-050P SiPMs, using Rhodorsil 47 V optical grease.

The size of the confinement region is primarily determined by the separation distances between scin- tillator detectors and the ^22^Na source. The source is placed 5 mm from the scintillator crystal under investigation. The reference scintillator detector is a further 40 mm on the opposite side from the source, unless otherwise stated. As the ^22^Na cylinder is not a point source, its finite size of 1 mm^3^ gives a minimum to the confinement region. For a source much closer to the scintillator detector under interest than to the reference detector, the confinement region will tend to the width of the source.

**Figure 4:**
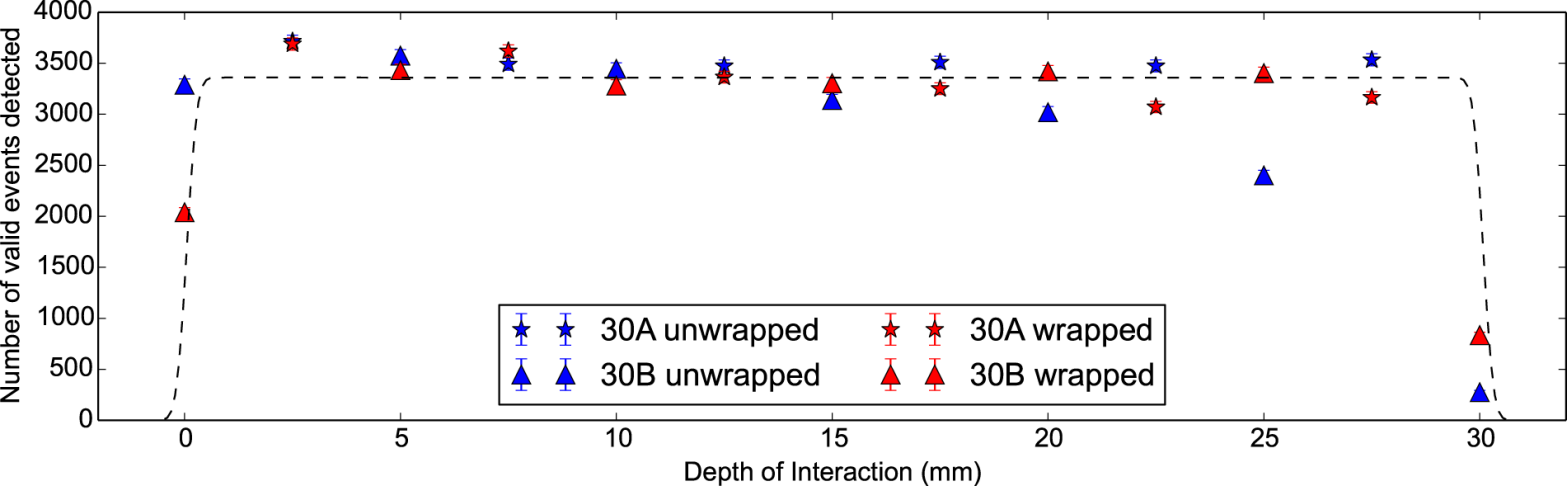
Number of valid *γγ* events detected against DOI for 30 mm LYSO:Ce scintil- lator crystals. The number of *γγ* events recorded with DOI for two Proteus 2×2×30 mm^3^ LYSO:Ce scintillator crystals. A uniform distribution convolved with a Gaussian distribution with FWHM of 1 mm is plotted as a dashed black line.

To determine the size of the confinement region we can exploit the fact that the scintillator detector will detect a fixed number of events per unit time if the volume of scintillator crystal does not change. Therefore for the same measurement and same confinement region we can assume a uniform number of events, regardless of DOI. Furthermore if the confinement region passes outside the scintillator crystal, the number of *γγ* events will drop until electronic collimation prevents any correlations from being detected. In this we assume good alignment of the scintillator crystal with respect to the central axis of the coincidence apparatus. We represent this described behaviour as a convolution between a uniform distribution and a Gaussian distribution. The uniform distribution has a width corresponding to the scintillator crystal length and an amplitude corresponding to the mean number of detected *γγ* events. The FWHM of the normal distribution corresponds to the confinement region; In this case taken as 1 mm. As shown in figure 4 as a black-dotted line this is a valid assumption for our apparatus on the provision the scintillator crystal is properly aligned.

### 30 mm LYSO:Ce CTR Results

Two identical polished Proteus LYSO:Ce 2×2×30 mm^3^ scintillator crystals, which we will refer to as 30A and 30B, are measured at DOI values between 0 and 30 mm in 2.5 mm increments alternating between the two scintillator crystals. This is to determine the contribution, if any, of systematic errors introduced by differences in coupling, alignment and surface finish. Each measurement is repeated with and without PTFE tape. The PTFE tape covers all faces except that in contact with the photodetector. Several tightly-bound layers of PTFE are used to increase adhesion with the scintillator crystal. Measurements with complete wrapping will referred to as the wrapped configuration. Likewise no wrapping is referred to as the unwrapped configuration. Each measurement was taken for 54 minutes each. As in the standard coincidence measurements, the first 5 minutes prior to data collection are ignored to ensure temperature stability within the sealed apparatus box.

**Figure 5:**
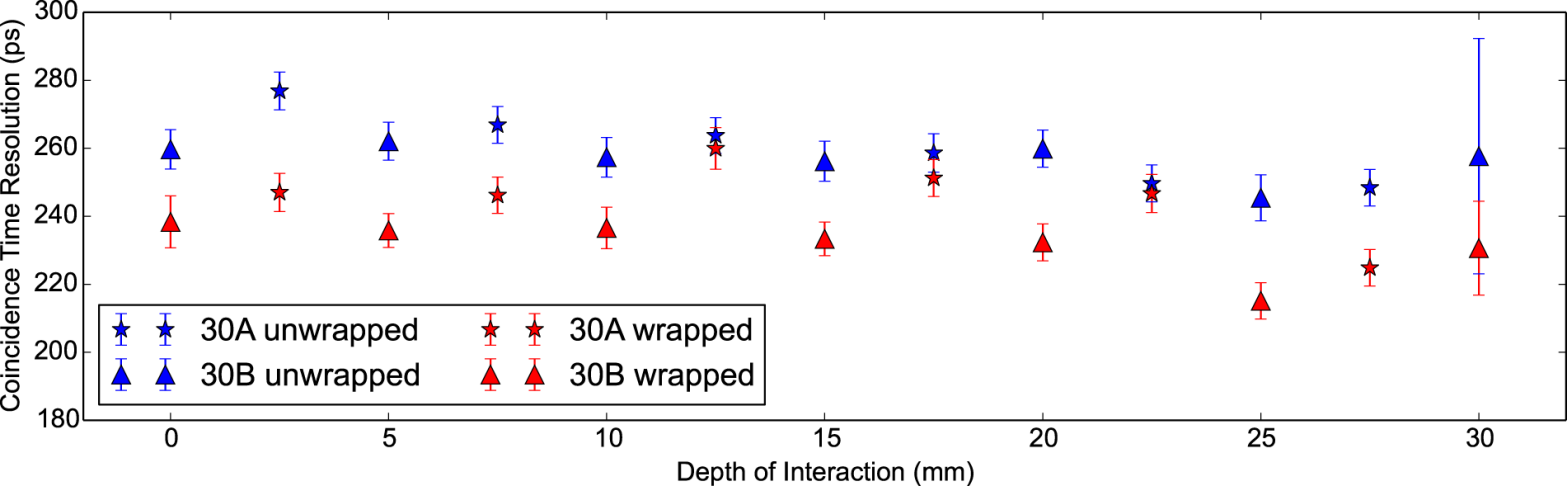
CTR against DOI for 30 mm LYSO:Ce scintillator crystals. Coincidence time resolution (CTR in ps) versus the depth of interaction (DOI in mm) for two Proteus 2×2×30 mm^3^ LYSO:Ce scintillator crystals in the wrapped and unwrapped configurations. Measurements are alternated with increasing DOI to check for any systematic error introduced by individual LYSO:Ce scintillator crystals.

In figure 5 the CTR (in ps) against DOI (in mm) per sample and configuration is given. In table 4 the values given for the timing and energy performance are averaged across the DOI. Firstly we note that no clear relationship between CTR and DOI is visible. The reduced chi-squared fit shows values close to unity for fitting to the weighted mean, indicating no relationship between CTR and DOI in both crystals and configurations. Secondly the CTR measurements from the wrapped configuration are consistently better than those from the unwrapped. The differences being 15±3 ps and 25±6 ps for 30A and 30B respectively. This difference is much smaller than that which we would expect in the standard CTR measurement. For instance it is seen in [Table 4] [14] that the difference in the CTR between wrapped and unwrapped configurations is approximately 33%. The differences for 30A and 30B are 6±1% and 10±2%. This implies that knowledge of the excitation position within the standard coincidence apparatus for an unwrapped scintillator crystal would reduce the measured CTR by at least 23%. We would predict this behaviour is due to a reduction in the variance of the photon travel time to the photodetector across multiple gamma ray photon detections. With DOI information, and limited diffusion in a polished unwrapped scintillator crystal, the photon travel time variance will be low.

**Table 4:** Mean values for energy and timing performance of 30

SampleB configuration	Right Energy Resolution (%)	Valid *γγ Events*	Detected *γγ* Events	Delay Peak Centroid (ps)	CTR (ps)	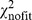
30A unwrapped	15.76±0.02	3532±24	64342±104	316.9±0.7	260.7±2.2	3.3
wrapped	13.84±0.02	3360±24	65132±104	321.4±0.7	246.0±2.3	3.6
30B unwrapped	17.17±0.03	2735±20	56030±89	306.0±1.3	256.9±5.4	0.6
wrapped	13.98±0.02	2816±20	60229±93	293.6±0.7	231.7±2.8	1.7

Results are grouped by sample and configuration. 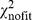 refers to reduced chi-squared of fitting the weighted mean to the data.

### 30 mm LYSO:Ce Additional timing and energy results

**Figure 6:**
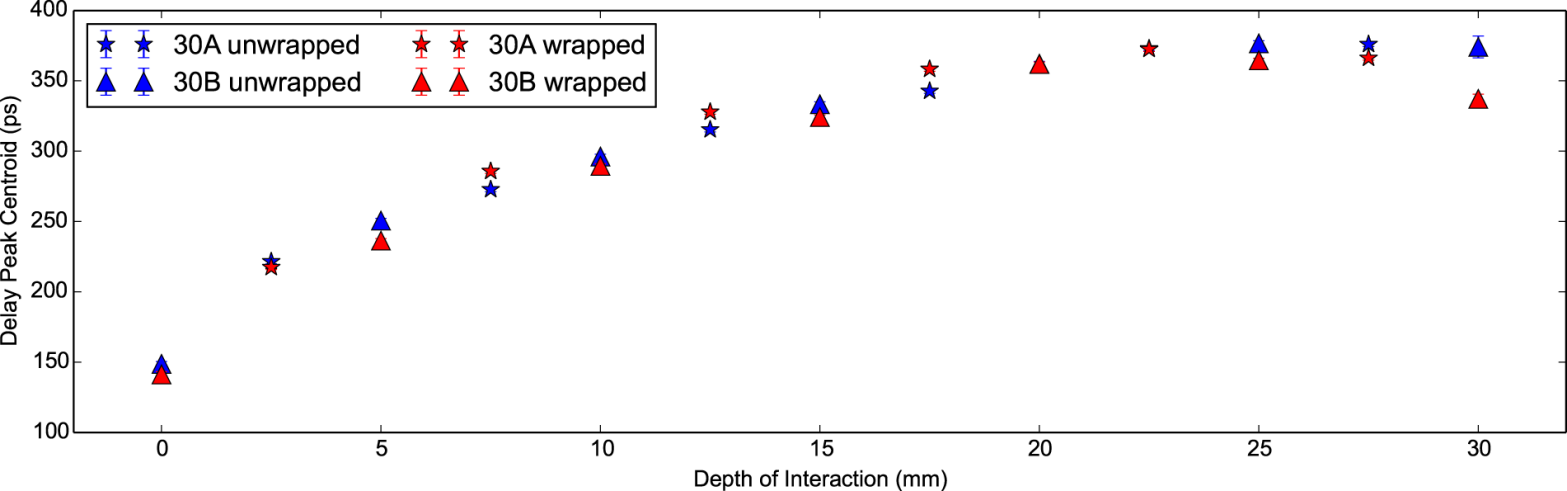
Delay peak centroid against DOI for 30 mm LYSO:Ce scintillator crystals. The delay peak centroid versus the depth of interaction plotted for two Proteus 2×2×30 mm. LYSO:Ce scintillator crystals in the unwrapped and wrapped configurations. The plateau is seen in all measurements at a DOI of approximately 20 mm.

In the coincidence apparatus measurements additional properties are recorded; namely the delay peak centroid, the light output and the energy resolution. In figure 6 the shift in the delay peak position with increasing DOI is plotted. It can be seen that a plateau is reached for both configurations at 20 mm. In [19] this plateau is attributed to the travel time between photons travelling ‘towards’ and ‘away’ from the photodetector approaching equality. As the electronic trigger from the discriminator is tuned as close to the beginning of the signal as possible this cannot come before generated light has physically travelled from its emission position to the photodetector. This is supported by the total shift seen of 150 ps to 370 ps corresponding to an approximate physical distance of 30 mm in L(Y)SO.

**Figure 7:**
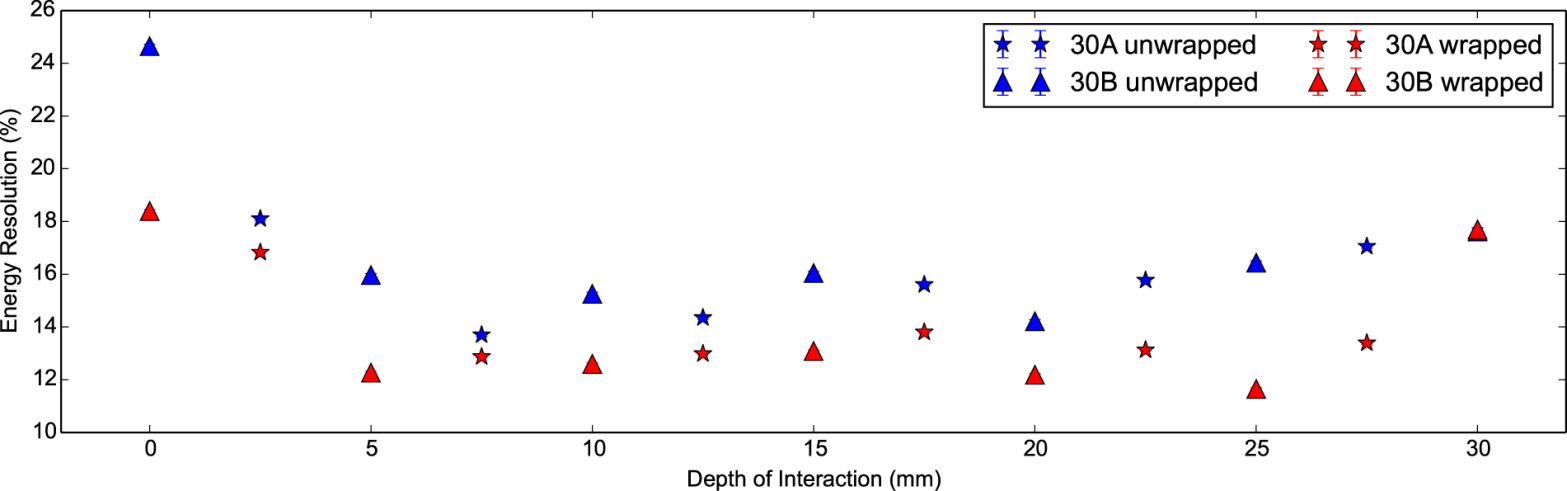
Energy resolution against DOI for 30 mm LYSO:Ce scintillator crystals. The energy resolution (%) versus the depth of interaction plotted for two Proteus 2×2×30 mm^3^ LYSO:Ce scintillator crystals in the unwrapped and wrapped configurations. For a DOI greater than 5 mm, the mean energy resolutions are 16.52±0.02% and 13.92±0.01% for the unwrapped and wrapped configurations respectively.

**Figure 8:**
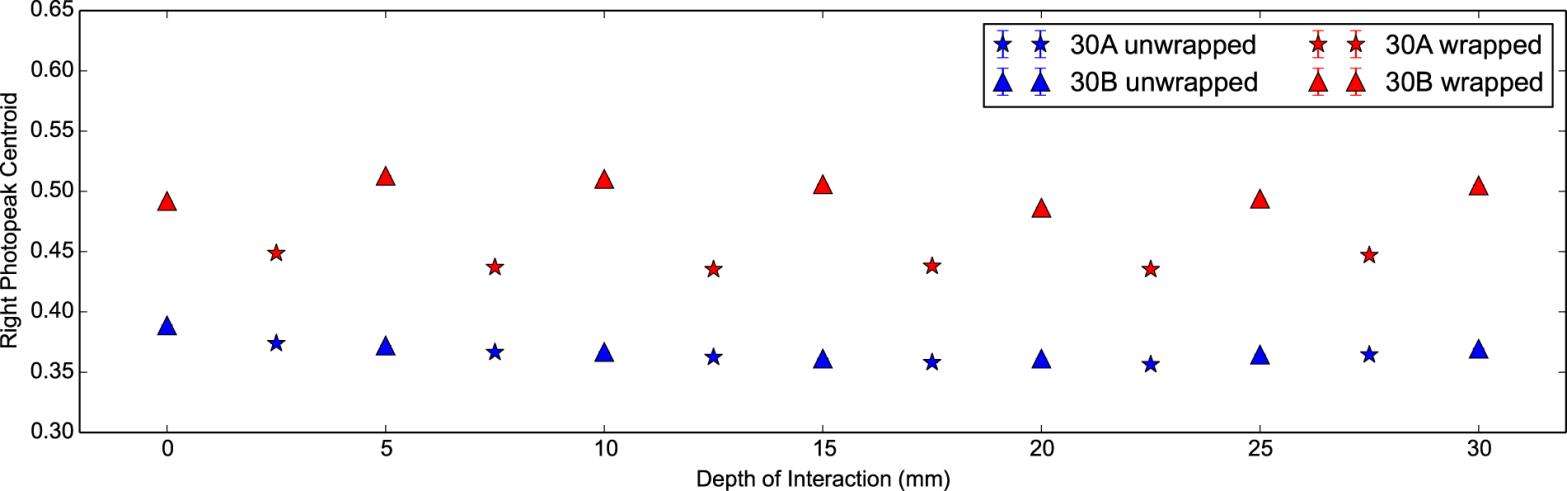
Light output against DOI for 30 mm LYSO:Ce scintillator crystals. The right photopeak centroid versus the depth of interaction for two identical LYSO:Ce scintillator crystals of shape 2×2×30 mm^3^ in the wrapped and unwrapped configurations. The right photopeak centroid corresponds to the absolute light output of the scintillator detector under investigation.

In figures 7 and 8 we can see the energy resolution and right photopeak centroid of the scintillator detector with DOI. The latter corresponds to the absolute light output arriving at the photodetector. Firstly we see the light output and energy resolution are better in the wrapped configuration compared to the unwrapped for both scintillator crystals as expected. For a DOI greater than 5 mm, the mean energy resolutions are 16.52±0.02% and 13.92±0.01% for the unwrapped and wrapped configurations respectively. Secondly we notice that the wrapped measurements show a systematic variation in the photopeak centroid, most likely due to differences in wrapping or coupling. Interestingly this pattern is also observed in figure 5 showing a poorer CTR for the ‘30B Wrapped’ compared to the ‘30A Wrapped’. Thirdly we notice that the light output is about 20% higher in the wrapped configuration with a minor drop off in both configurations with increasing DOI. We attribute this to longer path lengths through the scintillator crystal at higher DOI and therefore a greater chance of escape via Lobe reflection [20] or losses via absorption.

In all measurements the energy resolution is seen to be at its poorest for low DOI values, despite a weak variation seen in the light output. This indicates a broadening of the photopeak at low DOI values as no large change in the absolute light output is observed. Given that this broadening occurs only at low DOI this implies the cause is due to the geometry of the scintillator crystal. Specifically at low DOI, the solid angle of generated light reaching the photodetector without interacting with the side faces is high. As the photopeak is not seen to shift, thus the same light output in each measurement, we can see no saturation effects due to the SiPM. Therefore the energy resolution degradation at low DOI can be attributed solely to the confinement of gamma ray photon interactions near to the photodetector.

### 20 mm LSO:CeCa CTR Results

To determine to what degree the material and scintillator crystal length contributes to the timing and energy performance we repeat the 30 mm measurements with a 2×2×20 mm^3^ Agile Ca-co-doped LSO:Ce scintillator crystal. Additionally we consider a third ‘partially wrapped’ configuration; namely that we wrap the side faces but leave the face opposing the photodetector unwrapped. In doing so we expect to reduce the light output and thus the contribution from the backwards reflecting mode. Each measurement is collected for 90 minutes with an additional 5 minutes ignored at the beginning to ensure temperature stability. In doing so we wish to determine if the null relationship between the CTR and DOI is consistent across more potential variables to determine any weakness, if any, in our conclusions thus far.

**Figure 9:**
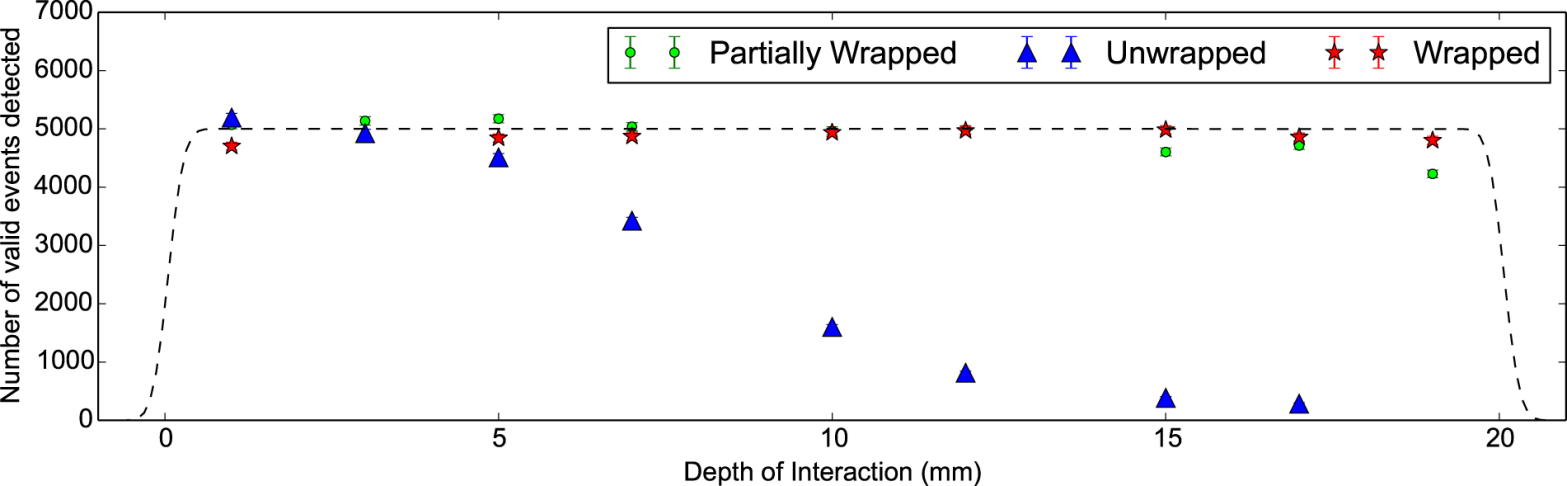
Number of valid *γγ* events detected against DOI for 20 mm LSO:CeCa scintillator crystals. Three configurations shown for a 2×2×20 mm^3^ LSO:CeCa scintillator crystal. In this we see that the partially wrapped and wrapped configurations shown good alignment, whereas the unwrapped shows poor. This will result in fewer events being collected and thus a larger error in higher DOI measurements.

**Figure 10:**
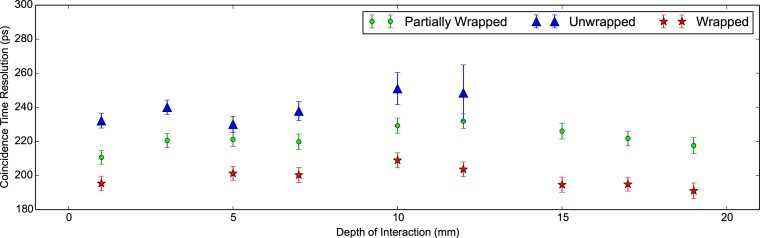
CTR against DOI for 20 mm LSO:CeCa scintillator crystals. The coincidence time resolution is plotted versus the depth of interaction for three configurations of a 2×2×20 mm^3^ LSO:CeCa scintillator crystal. Wrapped refers to covering all faces except that in contact with the photodetector in PTFE tape. Partially wrapped has only the side faces covered in PTFE tape leaving the face in contact and opposite the photodetector unwrapped. Unwrapped refers to no covering.

In figure 9 the number of detected *γγ* pairs is constant with DOI except for the unwrapped configu- ration. In this case we see a gradual drop off in the number of *γγ* events recorded with increasing DOI due to poor vertical alignment of the scintillator crystal. As the DOI is increased, the confinement region will drift outside the scintillator crystal and thus lead to a reduced number of *γγ* events detected in the 90 minutes per measurement. In figure 10 we see that this results in an increasingly larger error in the CTR until not enough events are collected to accurately determine the value at all. Even so, we find that poor alignment, whilst degrading the error in the measurement, does not introduce a systematic shift into the CTR. Thus as long as the number of *γγ* events collected is high then alignment is not a critical parameter.

In figure 10 we see the CTR improves (as in decreases) with increasing amounts of wrapping by 20 ps; from unwrapped, to partially wrapped and finally to wrapped. This is due to increased light output. These measurements have lower individual CTR errors than the previous 30 mm measurements, likely due to a greater number of *γγ* events recorded. These being approximately 5000 for LSO:CeCa compared to 3400 for LYSO:Ce, where these numbers are taken from figures 9 and 4 respectively. As the volume of the confinement region within the scintillator crystal is the same in both measurements this difference is primarily due to the difference in measurement time.

From table 5 the mean CTR for the wrapped configuration is 199±2 ps. In comparison an identical scintillator detector has a CTR of 176±7 ps for the standard coincidence measurement given in [Table 2] [13]. From table 3, for an equivalent 2×2×20 mm3 Proteus LYSO:Ce scintillator crystal a CTR of 202.7±4.0 ps in standard coincidence is observed. Therefore we see that LSO:CeCa is a superior material to that of LYSO:Ce. We also see that the CTR is worse in the DOI timing coincidence apparatus than in the standard. This conclusion is in agreement with the 30 mm measurements where we see values of 209.6±3.7 ps for the standard coincidence and 231.7±2.8 ps in the DOI coincidence apparatus. These are the two closest values from their respective experiments, indicating a minimum degradation of 10±2%. As the components and SiPM parameters are the same in both the standard and DOI coincidence apparatus, we would conclude this difference is due to the confinement region.

**Table 5:** Mean values for energy and timing performance of 20

Configuration	Right Energy Resolution (%)	Valid *γγ Events*	Detected *γγ* Events	Delay Peak Centroid (ps)	CTR (ps)	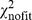
Unwrapped	19.99±0.03	3412±24	71805±109	148.9±0.9	240.0±3.5	1.1
Partially Wrapped	17.27±0.02	4879±23	83250±96	162.9±0.4	222.1±1.5	2.0
Wrapped	14.09±0.01	4872±25	86032±104	136.1±0.4	198.8±1.5	1.5

Results are grouped by sample and configuration. 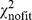 to fitting the weighted mean to the data.

### 20 mm LSO:CeCa Additional timing and energy results

**Figure 11:**
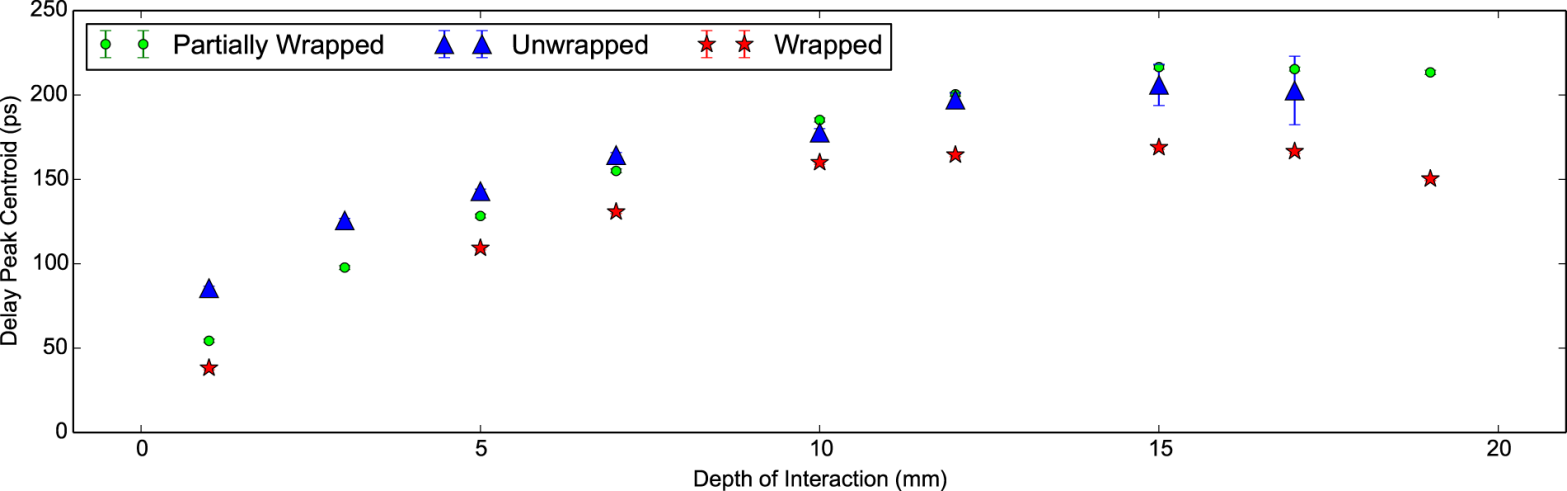
Delay peak centroid against DOI for 20 mm LSO:CeCa scintillator crystals. The delay peak position with depth of interaction is plotted for a LSO:CeCa scintillator crystal of shape 2×2×20 mm^3^ for the unwrapped, partially wrapped and wrapped configurations.

In figure 11 the delay peak position with DOI is seen to possess the same plateau as observed in the 30 mm measurements. In this case the plateau is reached close to 10 mm for the wrapped measurements. Again the peak to peak range in the delay peak centroid is comparable to the scintillator crystal length. We can conclude therefore that the shift is predominantly dependent upon the geometry of the scintillator crystal.

**Figure 12:**
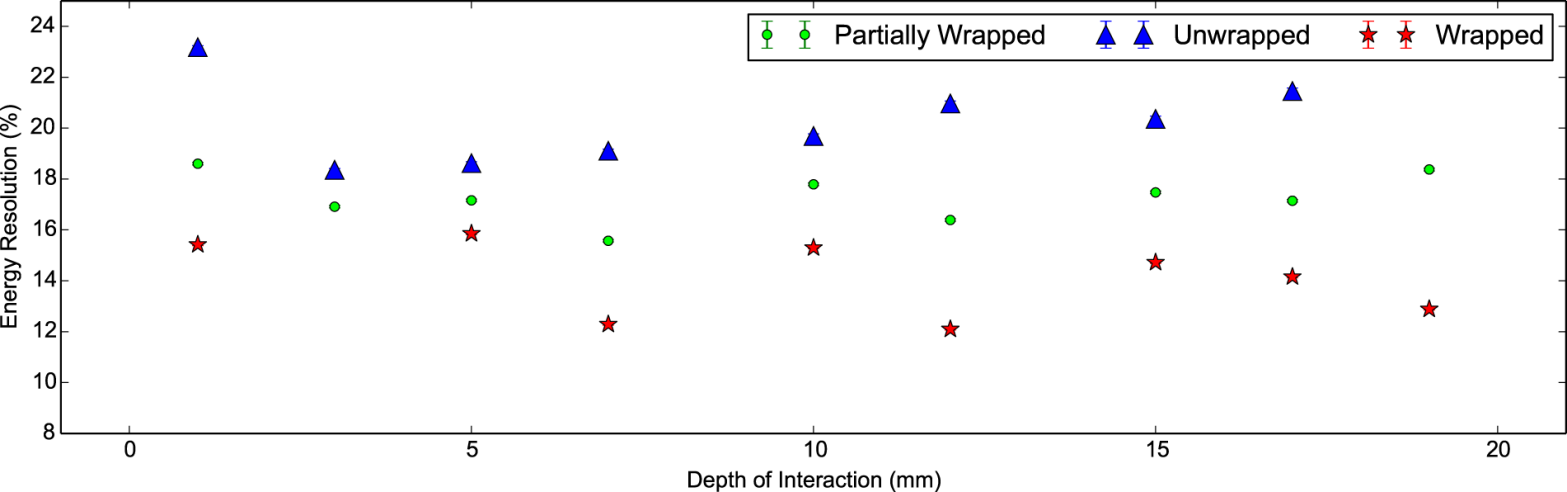
Energy resolution against DOI for 20 mm LSO:CeCa scintillator crystals. The energy resolution (%) for a LSO:CeCa scintillator crystal of shape 2×2×20 mm^3^ for the unwrapped, partially wrapped and wrapped configurations.

**Figure 13:**
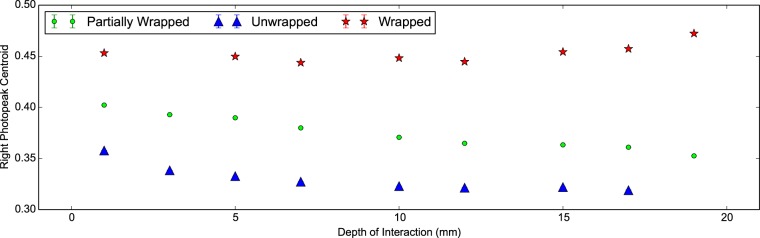
Light output against DOI for 20 mm LSO:CeCa scintillator crystals. The right photopeak centroid plotted against the depth of interaction for a LSO:CeCa scintillator crystal of shape 2×2×20 mm^3^ for the unwrapped, partially wrapped and wrapped configurations. The photopeak centroid corresponds to the light output of the scintillator detector.

In figures 12 and 13 we see the energy resolution and light output of the 20 mm measurements with DOI. As expected the wrapped measurements demonstrate the lowest energy resolution and the highest light output. At low DOI the energy resolution is at its poorest and the light output is at its highest, regardless of configuration. This implies light transport through the scintillator crystal leads to losses. The unwrapped configuration shows the largest drop in light output with DOI. Similarly in the partially wrapped configuration light is lost at increasing DOI. We conclude the predominant loss of light in both configurations is scattered light leaving through the face opposite the photodetector.

## Discussion

Our results indicate at most a weak dependence of the CTR upon the DOI. We conclude that the weight of evidence presented in the paper lends itself to the conclusion that no relationship, within experimental error, was found between the coincidence time resolution and depth of interaction. This result is in agreement with [21] for polished scintillator crystals. The mean CTR recorded from the DOI coincidence apparatus was consistently poorer than equivalent measurements performed using the standard coinci-dence apparatus, despite the same equipment being employed. In additional measurements, given with the full data in supplementary material, we conclude this is not due to the threshold voltage. Crucially no difference between the standard and DOI measurements was seen in the reference scintillator detector CTR. We conclude that the confinement region for long wrapped scintillator crystals has a negative effect upon the CTR. As the main physical change between the standard and DOI measurements is the variance in light transport within the scintillator crystals, it is logical to conclude this is the property responsible.

The results presented in [Figure 8] [19] show the delay peak centroid with DOI shifting in the same manner as presented in figures 6 and 11. Furthermore in [19] the gradient of the delay peak centroid with DOI is presented as an effective refractive index such that *n  =  mc* where *m* is the gradient of the fitted line (in SI units) and *c* is the speed of light. For the 30 mm measurements, fitting to a subset of delay peak centroid data with DOI below 20 mm, we find 3.6±1.5 and 3.4±1.2 for the unwrapped and wrapped configurations respectively. For the 20 mm measurements, fitting to a subset of delay peak centroid data with DOI below 10 mm, we find 3.9±1.0, 5.0±0.6 and 4.4±1.1 for the unwrapped, partially wrapped and wrapped configurations respectively. In [19] a value of 3.9 is presented for polished scintillator crystals in good agreement with our calculated values for the effective refractive index. Of note is the higher value for the partially wrapped configuration. In this case, photons arriving at the face opposite the photodetector at shallow angles will escape. Therefore a significant portion of photons in the ‘backward propagating’ mode will reflect from the rear at higher angles and thus take longer on average to reach the photodetector. The curve shape is predominantly attributed to finite propagation time of information through the scintillator crystal. Given that the plateau occurs at high DOI we would conclude that the variation in the time between the forward and backward modes becomes negligible and as such no longer moves the delay peak.

The light output and energy resolution are seen in both the 20 mm and 30 mm measurements to degrade weakly with increasing DOI. This behaviour is attributed to losses from a higher path length through the scintillator crystal. The severe penalty in the energy resolution at very low DOI is primarily due to confinement of gamma ray photon interactions near to the photodetector.

## Supporting Information

Figure S1 **CTR against DOI for single 30** **mm LYSO:Ce scintillator crystal.** Coincidence time resolution (CTR in ps) versus the depth of interaction (DOI in mm) for a Proteus 2×2×30 mm3 LYSO:Ce scintillator crystal wrapped in PTFE. These measurements were conducted for 90 minutes each with the reference scintillator detector at 200 mm from the source.
(EPS)

Figure S2 **CTR against DOI for 20** **mm LYSO:Ce scintillator crystals.** Coincidence time resolution (CTR in ps) versus the depth of interaction (DOI in mm) for Proteus 2×2×20 mm3 LYSO:Ce scintillator crystals wrapped in PTFE. These measurements were conducted using the same parameters are those given in the paper for the 30 mm measurements.
(EPS)

Figure S3 **CTR against DOI for a single 20** **mm LSO:CeCa scintillator crystal with threshold voltage.** Coincidence time resolution (CTR in ps) versus the depth of interaction (DOI in mm) for an Agile 2×2×20 mm3 LSO:CeCa scintillator crystal wrapped in PTFE. The threshold voltage (in mV) of the right NINO discriminator is varied from the default of 80 mV to 200, 600 and 1000, to determine its contribution (if any) upon the timing performance. No variation with depth of interaction is seen. Some degradation with threshold voltage is observed as expected.
(EPS)

File S1 **Supporting Information.**
(ZIP)
