# Supplementary material for: Influence of Depth of Interaction upon the Performance of Scintillator Detectors
Source: PLoS One. 2014 May 29;9(5):e98177. doi: 10.1371/journal.pone.0098177 (PMC4038557; doi:10.1371/journal.pone.0098177)
Supplement: File S1 — Supporting Information. (ZIP) [file pone.0098177.s004.zip › supporting information/doipaper_supporting_information.pdf]

Support Information Legends

- **Figure S1. CTR against DOI for single 30mm LYSO:Ce scintillator crystal. (extended-ctrdoi.eps)**

  Coincidence time resolution (CTR in ps) versus the depth of interaction (DOI in mm) for a Proteus $2 \times 2 \times 30$ mm^3 LYSO:Ce scintillator crystal wrapped in PTFE. These measurements were conducted for 90 minutes each with the reference scintillator detector at 200 mm from the source.

- **Figure S2. CTR against DOI for 20mm LYSO:Ce scintillator crystals. (lyso20mm-ctrdoi.eps)**

  Coincidence time resolution (CTR in ps) versus the depth of interaction (DOI in mm) for Proteus $2 \times 2 \times 20$ mm^3 LYSO:Ce scintillator crystals wrapped in PTFE. These measurements were conducted using the same parameters are those given in the paper for the 30mm measurements.

- **Figure S3. CTR against DOI for a single 20mm LSO:CeCa scintillator crystal with threshold voltage. (threshold-ctrdoi.eps)**

  Coincidence time resolution (CTR in ps) versus the depth of interaction (DOI in mm) for an Agile $2 \times 2 \times 20$ mm^3 LSO:CeCa scintillator crystal wrapped in PTFE. The threshold voltage (in mV) of the right NINO discriminator is varied from the default of 80mV to 200, 600 and 1000, to determine its contribution (if any) upon the timing performance. No variation with depth of interaction is seen. Some degradation with threshold voltage is observed as expected.

Processed Data Description

There are three .csv files. These are

1. alldata.csv : all depth of interaction (DOI) measurements. In this file there are Proteus LYSO:Ce 2x2x20 mm3, 2x2x30 mm3 and Agile LSO:CeCa 2x2x20 mm3 measurements. 2x2x30 mm3 are conducted twice with different separation distances. Furthermore this file contains varying discriminator threshold values.

2. referencedata.csv : standard and DOI coincidence measurements on the 2x2x5 mm3 LSO:CeCa scintillator crystals. This file contains values for many various threshold and bias values. Use the values chosen in the paper.

3. standarddata.csv : Here lies all the measurements conducted on the Proteus LYSO:Ce 5x5xL mm3 (for L is 5, 10, 15, 20, 30 mm) on the standard coincidence apparatus. Again select for the correct threshold and bias for correct comparison.

In the notebooks folder the four IPython notebooks used to generate every table and figure used in the research article are presented. These notebooks are included to ensure clarity in how data is manipulated to produce the stated results.

Processed Data Column Key

Name	Column Description
0	index column (ignore)
LPloc	Left photopeak location
LPlocerr	Left photopeak location error
LPscale	Left photopeak scale
LPscaleerr	Left photopeak scale error
RPloc	Right photopeak location
RPlocerr	Right photopeak location error
RPscale	Right photopeak scale
RPscaleerr	Right photopeak scale error
RSPloc	Right secondary photopeak location
RSPlocerr	Right secondary photopeak location error
RSPscale	Right secondary photopeak scale
RSPscaleerr	Right secondary photopeak scale error
SampleA	Left scintillator crystal name
SampleB	Right scintillator crystal name
amplitude	Amplitude of delay peak
amplitudeerr	Amplitude of delay peak error
chisquared	Reduced chi squared error in delay peak fit
ctime	clock time of measurement
length	length of right scintillator crystal
location	Location of delay peak
locationerr	Location of delay peak error
mtime	machine time of measurement
numofsamples	number of valid gamma-gamma events in delay peak in measurement
scale	scale parameter of delay peak
scaleerr	scale parameter of delay peak error
totalevents	total number of gamma-gamma events in measurement
uniquename	names of each and every measurement.
configuration	wrapping, no wrapping, partial wrapping
specialkey	keyword corresponding to measurement set
DOI	depth of interaction in mm
TimeResolution	time resolution in ps
TimeResolutionerr	time resolution in ps error
CTR	coincidence time resolution in ps
CTRerr	coincidence time resolution in ps error
ERright	Energy resolution in right scintillator detector
ERrighterr	Energy resolution in right scintillator detector error
ERleft	Energy resolution in left scintillator detector
ERlefterr	Energy resolution in left scintillator detector error

Tables

Table 1. Mean values for energy and timing performance of extended 30 mm DOI measurements. Results are grouped by sample and configuration. χ^2_{nofit} refers to reduced chi-squared of fitting the weighted mean to the data.

SampleB configuration	Right Energy Resolution (%)	Valid $\gamma\gamma$ Events	Detected $\gamma\gamma$ Events	Delay Peak Centroid (ps)	CTR (ps)	χ^2_{nofit}
30B wrapped	13.29±0.02	2713±20	78683±106	16.3±0.7	246.4±2.3	0.6

Table 2. Mean values for energy and timing performance of Proteus LYSO:Ce 20 mm DOI measurements. Results are grouped by sample and configuration. χ^2_{nofit} refers to reduced chi-squared of fitting the weighted mean to the data.

SampleB configuration	Right Energy Resolution (%)	Valid $\gamma\gamma$ Events	Detected $\gamma\gamma$ Events	Delay Peak Centroid (ps)	CTR (ps)	χ^2_{nofit}
20A wrapped	15.78±0.04	1394±17	41717±91	206.0±1.6	227.7±6.7	0.4
20B wrapped	14.47±0.02	1715±13	47565±69	221.2±0.9	229.3±3.9	0.9

Table 3. Mean values for energy and timing performance of a single Proteus LYSO:Ce 20 mm DOI measurements with threshold voltage of the right NINO discriminator. Results are grouped by sample and configuration. χ^2_{nofit} refers to reduced chi-squared of fitting the weighted mean to the data.

Threshold (mV)	Right Energy Resolution (%)	Valid $\gamma\gamma$ Events	Detected $\gamma\gamma$ Events	Delay Peak Centroid (ps)	CTR (ps)	χ^2_{nofit}
80	15.61±0.11	245±7	4883±31	346.7±3.1	308.3±10.5	2.1
200	15.78±0.05	1098±15	19073±62	550.2±1.6	337.4±5.6	1.1
600	16.48±0.06	1216±17	18268±68	797.4±1.8	357.8±6.0	1.4
1000	16.01±0.06	1197±17	17393±66	898.6±1.9	359.5±6.1	1.6
